# Wide‐Angle Domain Compatible Flexible Multispectral Stealth Metasurface for Visible Light, Laser, and Microwave

**DOI:** 10.1002/nap2.70323

**Published:** 2026-10-21

**Authors:** Lei Wang, Cuilian Xu, Ruichao Zhu, Hao Yang, Zuntian Chu, Jinming Jiang, Yuxiang Jia, Jiechu Liu, Qi Fan, Jiafu Wang

**Affiliations:** ^1^ Shaanxi Key Laboratory of Artificially‐Structured Functional Materials and Devices Air Force Engineering University Xi'an Shaanxi China; ^2^ SuZhou Laboratory Suzhou Jiangsu China

**Keywords:** 10.6 μm laser stealth, microwave stealth, multispectral compatibility, oblique incidence, visible‐light camouflage

## Abstract

As electromagnetic detection systems evolve toward multispectral integration and 0°–60° wide‐angle coverage, conventional multispectral stealth technologies suffer severe performance degradation at oblique incidence. Specifically, the 10.6 μm laser absorption peak shifts drastically at 30°, and X‐band microwave reflectivity exceeds 50% at 45°, failing stringent engineering demands. This work presents a wide‐angle‐compatible, flexible, multispectral stealth metasurface for visible light, laser, and microwave. It comprises a visible light camouflage layer (VCL), wide‐angle laser stealth layer (WLSL), and oblique‐incidence microwave absorption layer (OMAL). Large wavelength disparity across the three bands enables complete functional decoupling among layers. A resonance‐pre‐biased asymmetric Fabry–Pérot (F‐P) cavity maintains laser reflectivity below 20% at 60° incidence. A high‐duty‐cycle frequency selective surface (FSS) with double‐layer indium tin oxide (ITO) square rings eliminates the microwave transmission barrier from the WLSL, achieving > 85% absorptivity at 60°. Thin‐film interference modulates optical properties to match natural backgrounds for visible camouflage. Fabricated on PI/PC/PET flexible substrates via electron beam evaporation and laser etching, it offers conformal application potential and technical feasibility for batch manufacturing and cost optimization. This work breaks the angle‐sensitivity limitation of conventional designs and provides a feasible solution for wide‐angle multispectral stealth.

## Graphical abstract

A flexible, wide‐angle‐compatible multispectral stealth metasurface is proposed. It achieves visible camouflage and stable cooperative stealth of 10.6 μm laser and X‐band microwave across the 0°–60° incident range via layered wavelength decoupling.

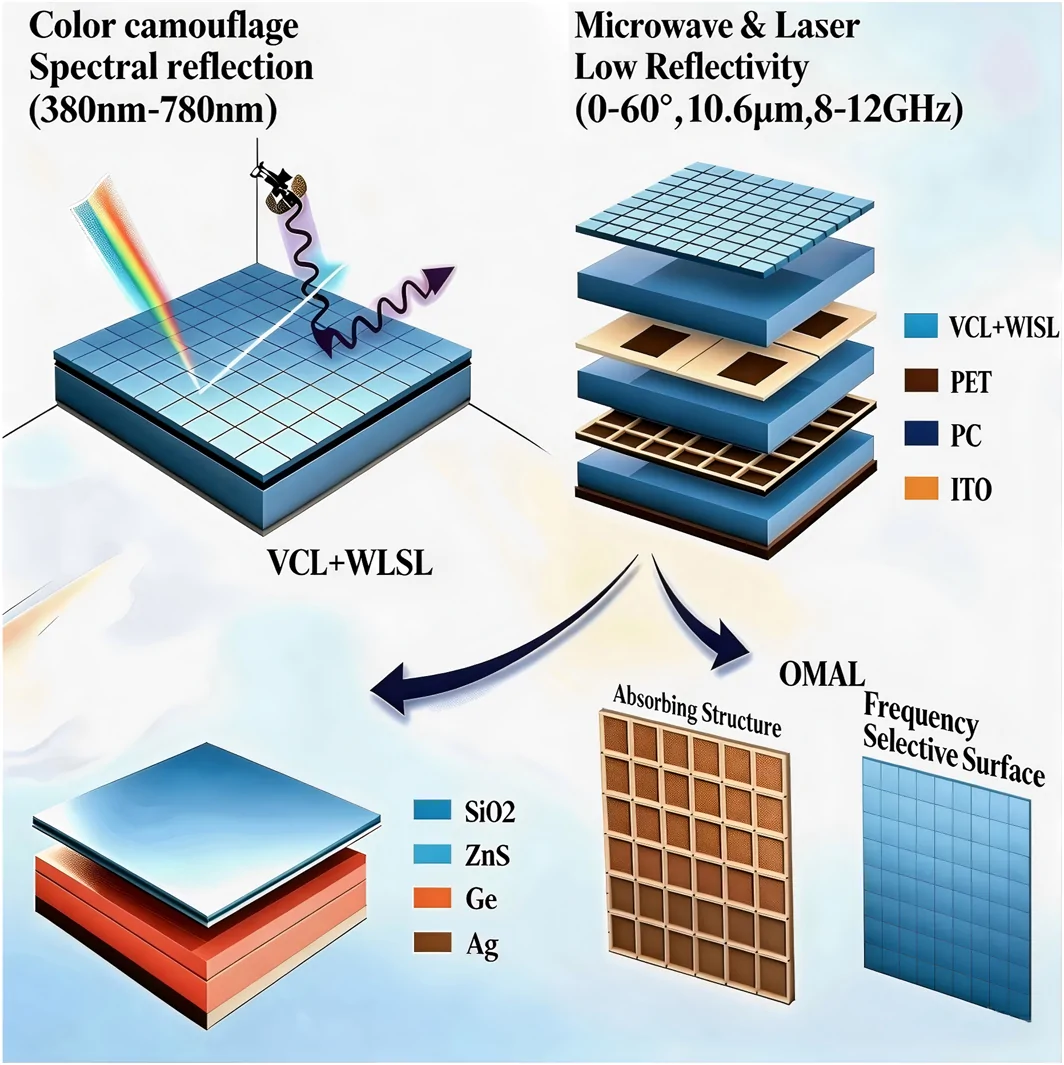

## 1 Introduction

With the rapid development of electromagnetic detection systems toward multispectral collaboration and wide‐angle domain coverage, modern multispectral photoelectric detection capabilities have achieved a qualitative leap [1, 2, 3]. Advanced detection systems can simultaneously acquire target information from visible light, infrared laser, and microwave bands, and realize all‐round, all‐weather target identification through multidimensional data fusion [4, 5, 6, 7, 8]. More critically, the detection angle range of modern systems has been extended from traditional normal incidence to 0°–60° wide‐angle domain, which poses unprecedented challenges to the stealth protection and survivability of engineering targets [9, 10, 11].

Traditional multispectral compatible stealth technologies face three inherent bottlenecks that are difficult to break through. First, there are severe functional conflicts between different stealth bands. Visible‐light camouflage requires precise spectral matching with the background, whereas laser and microwave stealth demand strong electromagnetic absorption at specific wavelengths, leading to complex structural design and mutual interference between functional layers [12, 13, 14, 15]. Second, the angle sensitivity problem is particularly prominent [16, 17]. Conventional structures suffer from significant absorption peak shift and performance degradation under oblique incidence. For 10.6 μm laser stealth, which is the core working band of CO_2_ laser‐based photoelectric detection and ranging systems, most existing designs can only maintain effective absorption within 0°–20° incidence range. When the incident angle exceeds 30°, the laser absorptivity drops below 60%, and completely fails at 45° incidence [18, 19, 20]. Third, most stealth structures rely on rigid substrates such as ceramics and epoxy resins, which lack conformal capability for complex curved surfaces of engineering equipment and carriers, severely restricting their practical engineering applications [21, 22].

To address these problems, extensive research has been conducted in recent years. For multi‐band compatible stealth, layered architectures have become the mainstream strategy [23, 24]. Feng et al. [25] proposed a hierarchical metamaterial realizing compatible stealth of 1.06 μm laser, 3–14 μm infrared, and 7–12.7 GHz microwave at normal incidence. Wang et al. [26] constructed an integrated visible‐light–laser–infrared–microwave stealth structure based on an asymmetric F‐P cavity, laying a foundation for multi‐band functional integration under normal incidence. However, most existing layered decoupling designs are optimized for normal incidence, without systematic consideration of multi‐band synergistic performance under wide‐angle oblique incidence. For wide‐angle stealth, microwave technologies are relatively mature, and relevant exploration has been extended to the optical band. Nazari et al. [27] inversely designed an aperiodic multilayer graphene absorber maintaining ≥ 90% mid‐infrared absorption under 0°–52° oblique incidence, but did not involve laser‐microwave compatibility. Lin et al. [28] proposed a *trans*‐scale hierarchical metasurface achieving microwave RCS reduction and high optical transmittance over 0°–60° incidence, yet its optical function focuses on transmittance regulation without targeted wide‐angle optimization for laser stealth. Song et al. [29] realized laser scattering stealth by modulating the phase difference between an F‐P resonant cavity and a chessboard ZnS metasurface, showing large‐angle application potential, but did not consider the wide‐angle absorption performance of the microwave band. Overall, these studies mostly focus on wide‐angle regulation of a single band and fail to establish wide‐angle synergy between laser and microwave bands.

Accordingly, current multispectral stealth research still faces notable technical bottlenecks, and a systematic solution for cooperative wide‐angle stealth of 10.6 μm laser and X‐band microwave remains lacking. First, most multi‐band integrated designs only optimize normal‐incidence performance, and the synchronous performance degradation across bands under oblique incidence remains unresolved, making it difficult to support wide‐angle multi‐band cooperative stealth. Second, few angle‐adaptive designs target wide‐angle 10.6 μm laser absorption—the most widely used wavelength in laser‐based photoelectric detection—as they mostly focus on optical transparency or mid‐infrared absorption regulation. Most importantly, no work has yet achieved wide‐angle cooperative stealth covering both 10.6 μm laser and X‐band microwave, the two core detection bands in modern multispectral detection scenarios, thus failing to meet the practical engineering requirements of complex wide‐angle detection.

To fill this research gap, this work proposes a wide‐angle‐compatible, flexible multispectral stealth metasurface integrating visible‐light camouflage, 10.6 μm laser stealth and X‐band microwave stealth. It features a top‐down hierarchical architecture consisting of VCL, WLSL, and OMAL. Table 1 compares the proposed design with typical multispectral stealth materials. This work delivers two core innovations. First, a resonance‐pre‐biased asymmetric F‐P cavity [30, 31] is proposed to realize stable 10.6 μm laser absorption over 0°–60° incidence. Second, a high‐duty‐cycle FSS with double‐layer ITO square rings [32, 33, 34] is designed to eliminate the microwave transmission barrier from the WLSL while maintaining stable X‐band microwave absorption under oblique incidence. Benefiting from the three‐order‐of‐magnitude wavelength difference across bands, functional decoupling and independent regulation of each layer are achieved with negligible interlayer crosstalk. Fabricated on PI/PC/PET flexible substrates via electron beam evaporation and laser etching, the design possesses flexible substrates and conformal application potential, as well as technical feasibility for batch manufacturing and cost optimization, showing broad application prospects in multispectral stealth engineering.

**TABLE 1:** Performance comparison of typical multispectral stealth materials.

Reference	Working bands	Incident angle range	Laser reflectivity	X‐band reflectivity	Visible function	Flexible substrate
[25]	1.06 μm laser/IR/microwave	Normal incidence	Intrinsic absorption	< 10%	—	No
[26]	Visible/laser/IR/microwave	Normal incidence	∼20% 1.06 μm	∼10%	Camouflage	Yes
[27]	3–5 μm mid‐IR	0°–52°	—	—	—	No
[28]	Visible/microwave	0°–60°	—	< 10%	High transmittance	No
[29]	Laser	Large angle potential	Scattering type	—	—	No
This work	Visible/10.6 μm laser/X‐band	0°–60°	< 20% 10.6 μm	< 15%	Camouflage	Yes

## 2 Results and Discussion

The design of multispectral stealth under oblique incidence should be centered on the synergistic satisfaction of stealth performance across multiple wavebands while ensuring excellent angular stability. For the 10.6 μm laser band and X‐band electromagnetic waves, effective suppression of reflected signals is achieved through structural optimization to reduce the identifiability of targets in these detection bands. In the 380–780 nm visible‐light range, the structural reflection spectrum needs to match the background environment to realize visual environmental integration. On this premise, targeted large‐angle incidence adaptation design is conducted for the laser and microwave bands to ensure the stable realization of target stealth performance within a wide range of incident angles.

To balance the performance requirements of each spectral range, a synergistic design scheme of a multilayer heterogeneous structure is adopted. Through the characteristic complementarity and parameter regulation of different functional layers, targeted performance optimization for each waveband is achieved. Meanwhile, a multispectral compatible system is constructed by leveraging the inherent wavelength differences between various wavebands, ensuring the synergistic realization of stealth functions across different wavebands without mutual interference.

### 2.1 Overall Design Logic

The multispectral stealth metasurface proposed in this paper adopts a top‐down hierarchical architecture, consisting of a VCL, a WLSL, and an OMAL in top‐to‐bottom order, as shown in Figure 1. The metasurface uses a 0.05 mm‐thick PI film and a 0.175 mm‐thick PET film as substrates, where the PI film supports the VCL and WLSL structures, while the PET film carries the OMAL wave‐absorbing structure. In terms of functional synergy, the VCL achieves color matching with environmental backgrounds, the WLSL realizes wide‐angle stealth for 10.6 μm laser, and the OMAL focuses on enabling high‐efficiency absorption of X‐band microwaves under oblique incidence.

**FIGURE 1:**
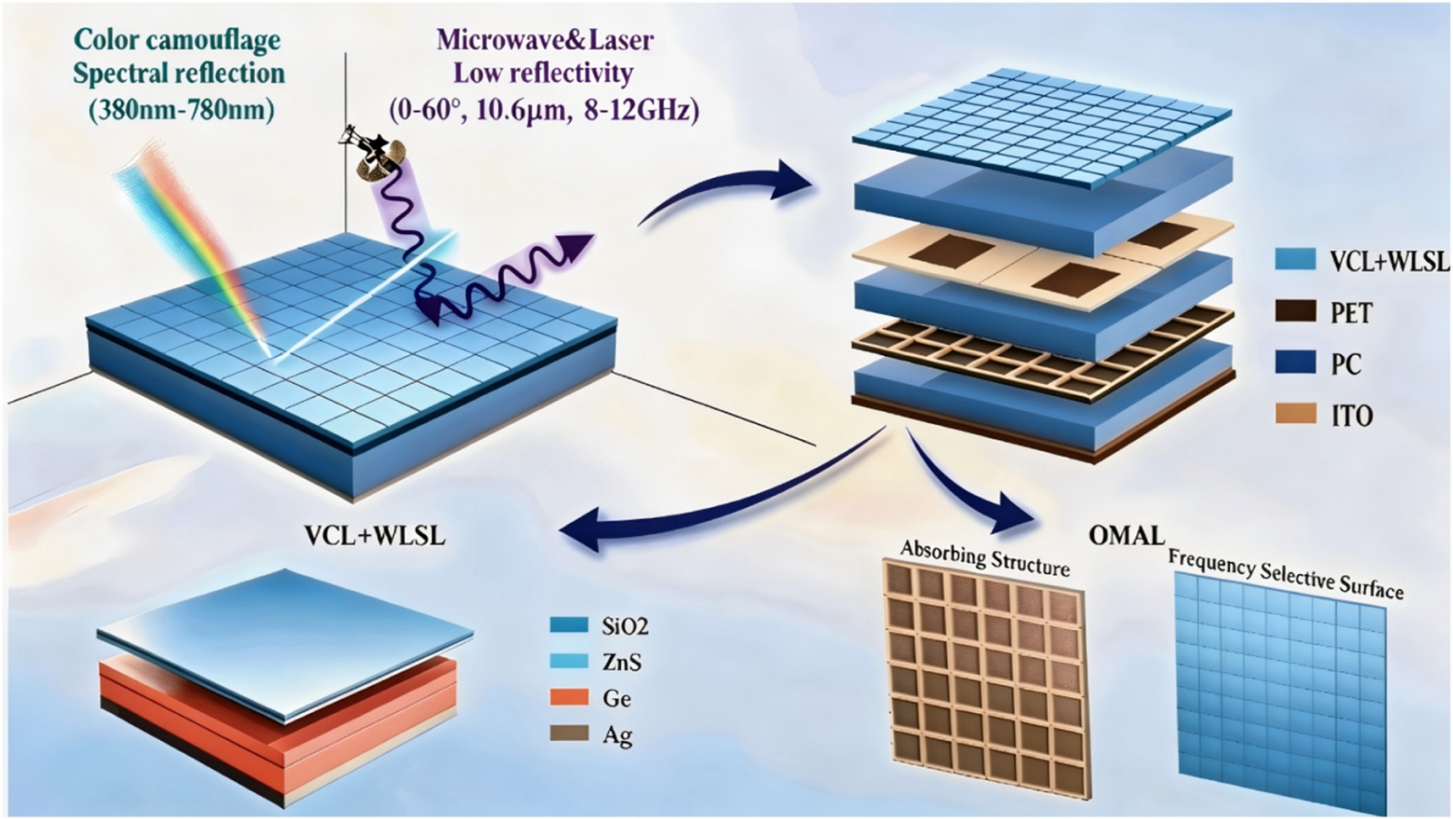
Schematic of the structure and working mechanism of the wide‐angle domain compatible flexible multispectral stealth metasurface for visible, laser, and microwave.

From the perspective of independent regulation, the crosstalk between functional layers can be effectively avoided based on the three orders of magnitude difference in wavelength scale among the visible‐light band 380 nm–780 nm, 10.6 μm laser, and X‐band microwaves. The VCL and WLSL with nanoscale thickness only respond to visible light and infrared laser. The OMAL with millimeter‐scale period only interacts with microwaves, and will not affect the optical performance of the upper layers. This wavelength‐scale decoupling mechanism provides a solid foundation for the independent design and integrated assembly of each functional layer.

For the structural optimization and fabrication sequence, this study adopts the core layer priority and functional layer sequential integration design strategy, which is determined based on the following considerations. First, the WLSL is the core component for solving the wide‐angle stealth bottleneck, and its performance directly determines the overall wide‐angle adaptability of the metasurface. Therefore, it is necessary to optimize the WLSL first to ensure that its 0°–60° laser absorption performance meets the design requirements. Second, the VCL is deposited on the surface of the WLSL, and its thickness adjustment will not affect the laser absorption performance of the WLSL. Therefore, the VCL can be designed and integrated after the WLSL optimization is completed. Third, the OMAL requires a laser etching process to form the FSS structure, which will penetrate the entire VCL and WLSL. Therefore, the OMAL must be fabricated after the VCL–WLSL composite layer is prepared, and the etching process parameters need to be strictly controlled to avoid damaging the underlying functional layers.

### 2.2 Design and Simulation of Wide‐Angle Laser Absorbing Layer

The WLSL serves as the core component for optimizing oblique incidence performance. In the structural design of the WLSL, an Ag/Ge/Ag/Ge multilayer film structure based on an asymmetric F‐P cavity was constructed in this paper. It is composed of a matching layer, a unidirectional transmission layer, a dielectric layer, and a reflective layer in a top‐down sequence, with the corresponding thicknesses denoted as *h*_1_, *h*_2_, *h*_3_, and *h*_4_, respectively. The final optimized parameters are determined as follows, *h*_1_ = 600 nm (Ge), *h*_2_ = 12 nm (Ag), *h*_3_ = 1230 nm (Ge), and *h*_4_ = 1000 nm (Ag).

This structure is derived from the classical F‐P cavity configuration (Ag/Ge/Ag). The classical structure can restrict the direction and frequency of oscillating light beams via optical feedback, and realize the absorption or radiation of infrared light within a specific range through parameter regulation. However, the ultrathin silver layer in the far‐infrared band has a small skin depth, which renders it technically challenging to achieve effective incidence of the target laser. To address this issue, this study optimizes the classical structure by introducing a Ge matching layer to adjust the interface impedance, improving the transmission efficiency of incident laser through the top ultrathin Ag layer and enabling efficient energy dissipation inside the F‐P cavity.

Under the calculation of the classical system, the electrical conductivity of the ultrathin Ag decreases due to the nanoscale size effect, with the electrical conductivity $\sigma^{\prime }$ = 4.0 × 10^7 ^S/m and the magnetic permeability $\mu \approx \mu_{0}$ = 4π × 10^−7^ H/m. Under the irradiation of 10.6 μm laser, the calculation of skin depth is shown in Equation (1) [35].

(1) $$\delta =\sqrt{\frac{2}{\omega \mu \sigma^{\prime }}}=\sqrt{\frac{2}{2\pi c/\lambda \cdot \mu \sigma^{\prime }}}\approx 13\text{nm}$$

where ω is the angular frequency of the 10.6 μm laser, μ is the magnetic permeability of Ag, and $\sigma^{\prime }$ is the corrected electrical conductivity of ultrathin Ag film.

To enable 10.6 μm laser light to pass through the top ultrathin Ag layer for unidirectional transmission, the thickness of the Ge matching layer is optimized. This optimization yields favorable impedance matching under normal incidence and improves energy coupling into the resonant cavity. For backward‐propagating reflected waves, the propagation direction is altered and the impedance matching condition is no longer satisfied. Such variation suppresses energy leakage from the cavity and enables effective confinement of laser energy within the cavity.

The bottom Ag layer, acting as the reflective layer, should have a sufficient thickness to ensure complete reflection without transmission loss. Together with the top Ag layer, it forms the two reflective surfaces of the cavity, confining the 10.6 μm laser within the cavity for absorption.

In addition, aiming at the problem that the F‐P cavity is prone to resonance wavelength blue shift and performance degradation under wide‐angle oblique incidence scenarios, performance optimization is realized via resonance wavelength pre‐biasing and bandwidth matching based on Equation (2). Its core physical significance lies in quantifying the blue shift amount of the resonance wavelength under different incident angles, and optimizing the Ge resonant layer thickness to ensure that 10.6 μm always falls within the high absorption region of the absorption peak.

(2) $$\left\{\begin{array}{l} \Delta \lambda =\lambda_{0}\cdot \left(1-\cos\;\theta_{t}\right)\\ n_{\text{air}}\;\sin\;\theta =n_{Ge}\;\sin\;\theta_{t} \end{array}\right.$$

where $\theta_{t}$ denotes the refraction angle of the laser in the Ge dielectric layer, θ denotes the laser incident angle, $n_{Ge}$ denotes the refractive index of Ge, Δλ denotes the blue shift of the resonant wavelength under oblique incidence, and $\lambda_{0}$ is the resonant wavelength of the F‐P cavity at normal incidence.

From the perspective of mechanism, when the laser is normally incident (θ=0°), the refraction angle $\theta_{t}=0{}^{\circ}$ and $\cos\;\theta_{t}=1$, so the blue shift amount Δλ = 0. At this time, the resonance wavelength is $\lambda_{0}$, which is determined by the optical thickness of the Ge resonant layer $n_{Ge}h_{3}$. However, when the laser is obliquely incident (θ>0°), the resonance wavelength will blue shift according to Equation (2). This causes the 10.6 μm laser to deviate from the resonance peak, thereby leading to issues such as reflectivity rise and performance degradation.

By optimizing the thickness of the Ge dielectric layer *h*_3_, the resonance wavelength at normal incidence $\lambda_{0}$ is pre‐biased to a value slightly longer than 10.6 μm. As the incident angle increases from 0° to 60°, the resonance wavelength gradually blue shifts toward 10.6 μm. Combined with the sufficient absorption bandwidth (about 1 μm) provided by the asymmetric cavity structure and material loss, 10.6 μm laser always falls within the high‐absorption region across the entire 0°–60° incident range. This resonance pre‐biasing mechanism, which compensates for the oblique‐incidence blue shift of the resonant wavelength, constitutes the core of wide‐angle laser stealth stability. The pre‐biasing design ensures that the target wavelength stays within the absorption band at all incident angles, while the inherent bandwidth of the cavity accommodates the resonance shift induced by angle variation. Without this synergistic design, wide‐angle laser stealth performance degrades rapidly beyond 30° incidence, as is characteristic of conventional F‐P cavity structures.

To verify the laser stealth performance of the designed structure, simulation analysis was carried out using the professional optical thin film software TFCalc. The simulation results are shown in Figure 2. Figure 2a presents the reflectance of 10.6 μm laser as a function of incident angle from 0° to 80°, covering S‐polarization, P‐polarization, and their average value. The results show that the reflectivity of 10.6 μm laser is stably maintained below 20% within the 0°–60° incident range, satisfying the engineering requirement for laser stealth. In terms of polarization dependence, S‐ and P‐polarized reflectance are highly consistent at small incident angles (0°–40°), with negligible difference. As the incident angle increases beyond 60°, the polarization difference gradually enlarges due to the polarization dependence of Fresnel reflection at the metal interface and the divergent blue shift of the resonant wavelength. Nevertheless, both polarizations meet the performance index within the designed 0°–60° working range. Figure 2c displays the reflectance spectra in the 5–16 μm wavelength range and the corresponding normalized squared electric‐field intensity (|E|^2^) distributions of 10.6 μm laser at 0°, 20°, 40°, 60°, and 80° incidence, respectively. The |E|^2^ distributions demonstrate that both S‐ and P‐polarized laser form stable standing wave patterns in the Ge resonant dielectric layer within the 0°–60° incident range. The upper and lower Ag reflective layers strictly correspond to the nodes of the standing waves where |E|^2^ approaches zero, while distinct field enhancement antinodes are formed inside the cavity. This confirms that laser energy is effectively confined in the asymmetric F‐P cavity for high‐efficiency absorption.

**FIGURE 2:**
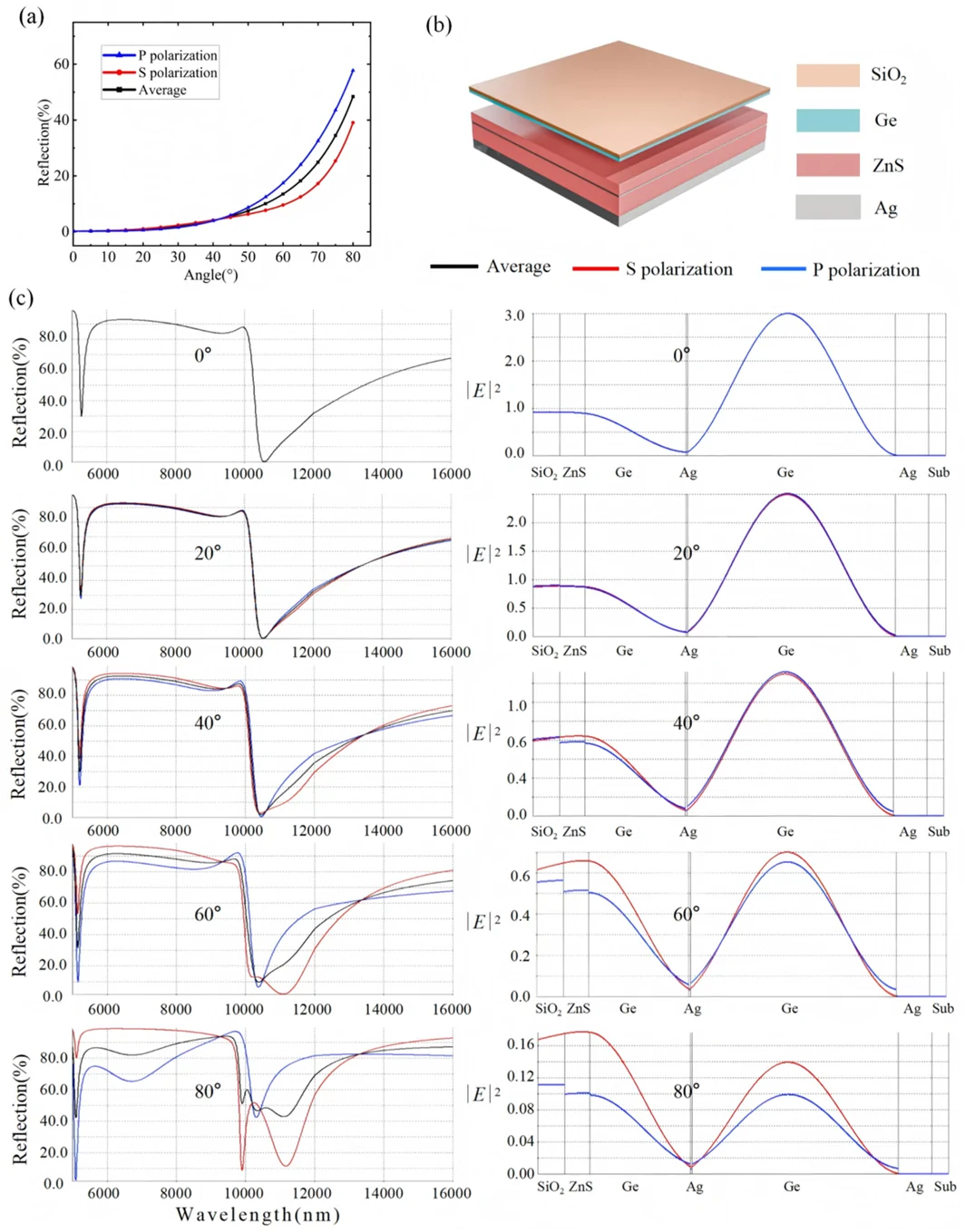
Laser simulation results. (a) Simulated 10.6 μm laser reflectivity as a function of incident angle (0°–80°) for S polarization, P polarization, and average value. (b) Schematic of the optical functional layer structure. (c) Simulated reflection spectra (5–16 μm) and normalized electric field intensity (|E|^2^) distributions at 0°, 20°, 40°, 60°, and 80° incidence.

In terms of the polarization dependence of the electric field, the |E|^2^ peak values for s‐ and p‐polarization are nearly identical at 0°–40° incidence, with only a small difference between the two. As the incident angle rises to 60°–80°, this gap gradually widens. The standing wave peak of s‐polarization is slightly higher than that of p‐polarization. This behavior arises from the higher Fresnel reflectivity of s‐polarization at the Ag interface, which leads to a higher quality factor of the resonant cavity. With increasing incident angle, the |E|^2^ peak value shows a monotonic decreasing trend. Even so, the integrity of the standing wave distribution remains well preserved within the 0°–60° range. This indicates that the resonance pre‐biasing design effectively compensates for the blue shift of the resonant wavelength under oblique incidence and mitigates performance degradation. It thereby verifies the wide‐angle laser stealth stability of the WLSL.

When the incident angle reaches 80°, an ultra‐large angle beyond the designed range, the |E|^2^ peak decreases sharply and the standing wave structure is severely distorted. Two main mechanisms account for this performance degradation. First, the nonlinearly increased blue shift of the resonant wavelength exceeds the bandwidth of the absorption peak. This causes severe mismatch of the resonant phase condition and weakened coherent absorption. Second, the reduced normal component of incident energy flux and the increased Fresnel reflection at the top Ag interface jointly reduce the effective energy coupled into the cavity. This further confirms that 0°–60° is the effective wide‐angle working range of the structure. From the perspective of engineering applications, the effective operating angles of mainstream photoelectric detection systems are mostly concentrated in the 0°–60° range. The performance range of this design can thus meet the requirements of typical application scenarios.

### 2.3 Design and Characterization of VCL

Deposited on the surface of the WLSL, the VCL serves as the core functional layer to realize color camouflage in the visible‐light band. Its structure consists of alternately stacked SiO_2_ (refractive index *n* ≈ 1.44) and ZnS (refractive index *n* ≈ 2.3) films. This layer modulates the reflection spectrum based on the thin film interference effect, thereby achieving color matching with different background environments, and the wavelength response characteristics of its reflection spectrum are directly determined by the film thickness.

The thin film interference principle is the core mechanism for the VCL to achieve precise color regulation. When visible light is incident on the alternately stacked SiO_2_/ZnS structure, reflected and transmitted light are generated at the interfaces of different film layers, and multiple coherent beams are superimposed on each other to form an interference effect. By precisely regulating the film thickness, constructive interference can be generated for visible light at specific wavelengths to enhance reflection, while other wavelengths are weakened due to destructive interference, finally enabling the structure to present the target camouflage color.

To simplify the simulation calculation and ensure the structural functionality, the thickness of the SiO_2_ layer is fixed at 160 nm in this paper. This layer simultaneously acts as a near‐infrared transition layer and a structural protective layer, with excellent wear and corrosion resistance. In the visible‐light band, since the underlying Ge layer is opaque, the reflection spectral characteristics of the structure are mainly determined by the thickness of the ZnS layer. Therefore, color matching with different natural backgrounds can be achieved by adjusting the thickness of the ZnS layer in the range of 20–200 nm.

Simulation analysis of the reflection performance of the VCL with different ZnS layer thicknesses was performed using TFCalc software. The results show that by changing the thickness of the ZnS layer, reflection enhancement peaks can be formed at different visible‐light wavelengths, thereby enabling the structure to present differentiated camouflage colors. Figure 3a presents the simulation results of visible‐light camouflage. For the vegetation background, the thickness of the ZnS layer is set to 45 nm. The structure exhibits a green color, with the corresponding CIE chromaticity coordinates of (0.26, 0.41). For the water background, the ZnS layer thickness is selected as 103 nm. The structure appears blue, with the CIE chromaticity coordinates of (0.23, 0.30). For the soil or desert background, the ZnS layer thickness is chosen as 125 nm. The structure presents a light yellow‐brown color, with the corresponding CIE chromaticity coordinates of (0.37, 0.41). The above parameter design ensures that the VCL can achieve color matching with typical natural backgrounds, thereby realizing effective visible‐light camouflage.

**FIGURE 3:**
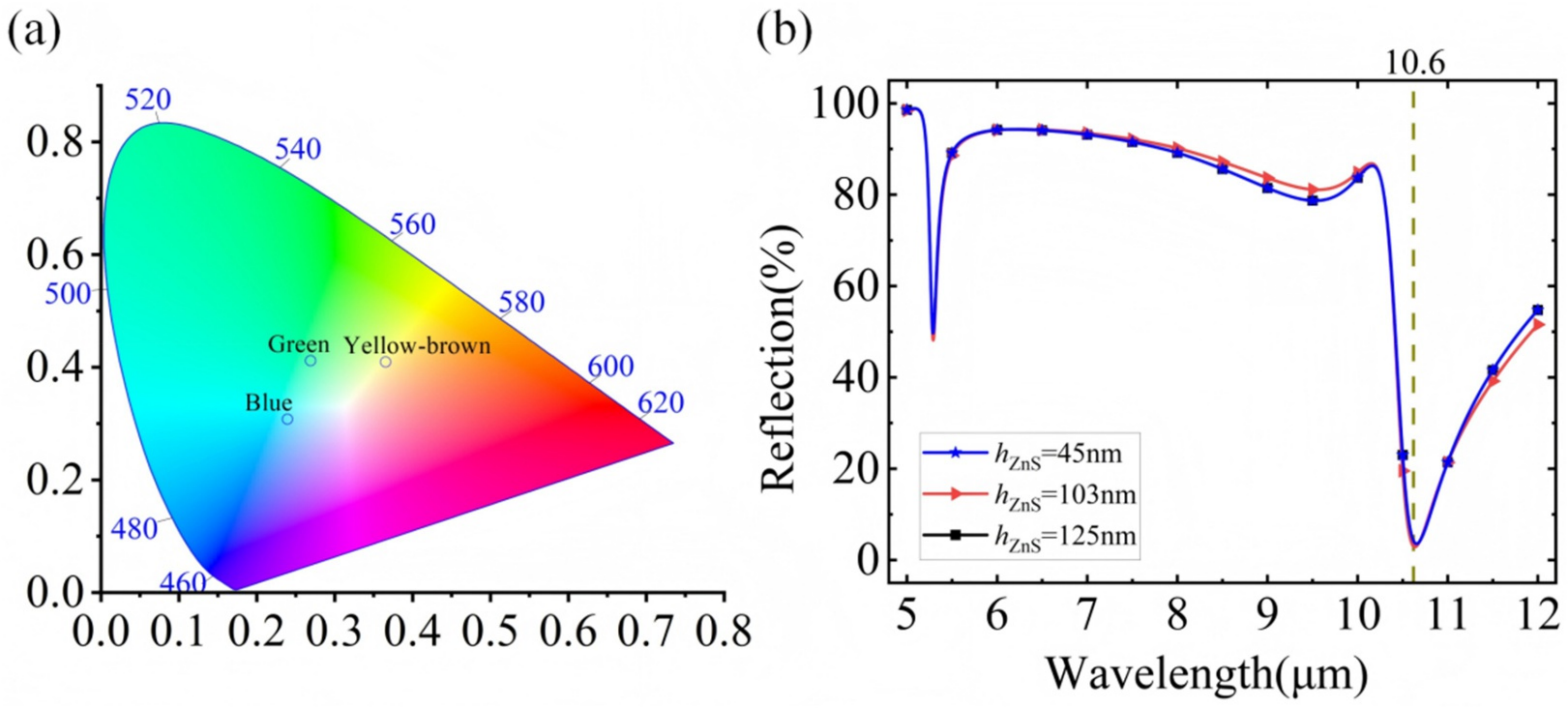
Simulation results of visible camouflage. (a) Simulated CIE 1931 chromaticity diagrams for three typical colors. (b) Effect of different ZnS layer thicknesses on the laser stealth performance of the WLSL.

To verify the interlayer compatibility between the VCL and WLSL, the influence of ZnS layer thickness adjustment on the laser stealth performance of the WLSL was systematically investigated. The simulation results in Figure 3b demonstrate that even when the ZnS layer thickness is adjusted across the full 30–300 nm range to match different background colors, the 10.6 μm laser reflectivity fluctuates within 2% under normal incidence and remains below 20% at 60° oblique incidence. This is because the ZnS layer, with a thickness of only tens to hundreds of nanometers, has negligible influence on the propagation of 10.6 μm infrared laser. No significant interference is introduced to the core laser absorption performance of the WLSL, demonstrating excellent interlayer synergy between the two functional layers.

### 2.4 Design and Simulation of the OMAL

The OMAL is the key functional layer for realizing oblique incidence stealth of X‐band microwaves. Its core design goal is to ensure efficient transmission of 2–14 GHz microwaves while achieving stable oblique incidence absorption of X‐band microwaves. From the perspective of structural design constraints, the VCL, as a dielectric layer, can realize low‐loss transmission of microwaves, but the underlying WLSL contains a metal layer, which will prevent microwaves from penetrating. Therefore, a FSS wave‐transparent structure fabricated by etching process is required to solve this problem. Based on this, the OMAL adopts a composite architecture of a FSS wave‐transparent structure and the double‐layer ITO square ring absorbing structure to realize the synergistic function of wave transmission and absorption.

The structural parameter design of the OMAL follows the principle of wave transmission performance priority and absorption performance adaptation. It mainly falls into two parts, namely the FSS wave‐transparent structure and the double‐layer ITO square ring absorbing structure. The FSS structure adopts a patch‐type design, which generates strong reflection at its resonant frequency, while electromagnetic waves with frequencies lower than the resonant frequency can achieve low‐loss transmission. According to the standing wave theory, its resonant frequency satisfies the following equation:

(3) $$f=c/2nw=\frac{c}{2w\sqrt{\left(\varepsilon_{r}+1\right)/2}}$$

where *w* is the patch width, $n=\sqrt{\varepsilon_{r}}$ is the refractive index of the dielectric material, *ε*_*r*_ is the relative permittivity of the dielectric, and *c* = 3 × 10^8^ m/s is the speed of light in vacuum. The refractive indices of the structures designed in this paper are all positive, so the smaller the patch size, the higher the corresponding resonant frequency, and the better the microwave transmission performance in the low frequency band. The core parameters of the FSS determined by simulation optimization are as follows: unit period *p*_1_ = 1.125 mm, patch side length *d*_1_ = 1.05 mm. These parameters ensure that the microwave transmittance within the target frequency band meets the design requirements.

This double‐layer ITO square ring absorbing structure is modified based on the classic ITO‐dielectric‐ITO sandwich absorption configuration. It achieves effective microwave absorption in the X‐band via impedance matching, and greatly reduces the polarization sensitivity under large oblique incidence angles. The upper ITO square ring is characterized by outer diameter *r*_1_, inner diameter *r*_2_, and sheet resistance *R*_1_. The lower ITO square ring has outer diameter *r*_3_, inner diameter *r*_4_, and sheet resistance *R*_2_. The sheet resistance of the bottom ITO layer is *R*_3_. In the proposed structure, the thickness of all PC layers is h1, and the thickness of PET layers is *h*_2_. The detailed core structural parameters are given as follows: unit period *p* = 9 mm, *r*_1_ = 9 mm, *r*_2_ = 6 mm, *r*_3_ = 2.5 mm, *r*_4_ = 1.5 mm, *R*_1_ = 25 Ω/sq, *R*_2_ = 10 Ω/sq, *R*_3_ = 5 Ω/sq, *h*_1_ = 2 mm, and *h*_2_ = 0.175 mm. This parameter design achieves efficient electromagnetic energy dissipation in the double‐layer ITO square ring region after microwaves propagate through the FSS structure.

Simulation verification of the microwave performance of the OMAL was carried out using CST Studio Suite 2023 electromagnetic simulation software. The simulation conditions include a frequency range of 2–18 GHz, TE and TM wave incidence modes, and the adoption of finite‐difference time‐domain (FDTD) solver. For oblique incidence performance, multiple groups of simulations in the incident angle range of 0°–60° were supplemented. The simulation results are shown in Figure 4.

**FIGURE 4:**
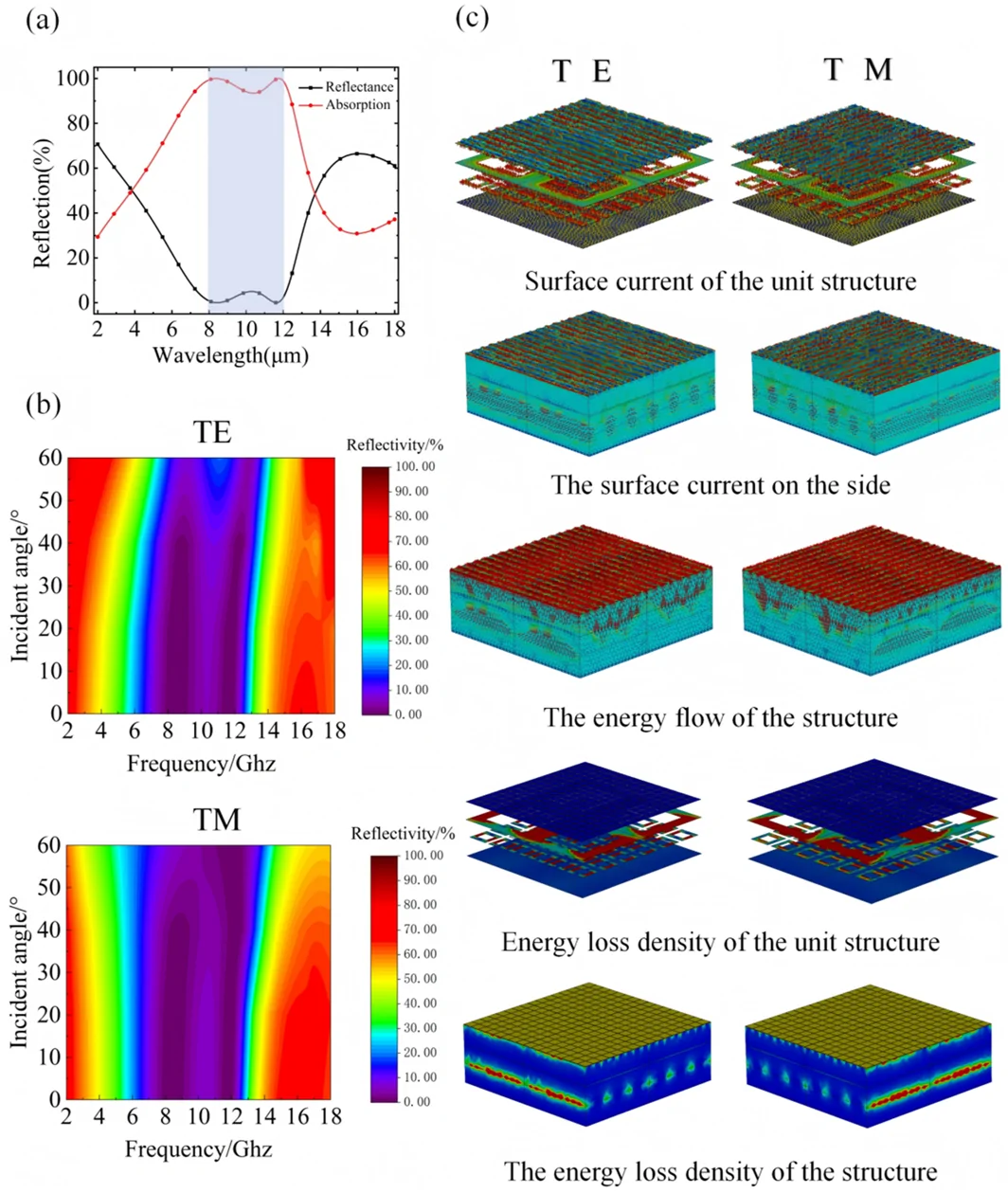
Microwave simulation results. (a) Microwave reflectivity simulation curve in 2–18 GHz. (b) Simulation results of microwave reflectivity at different incident angles. (c) Surface current, lateral surface current, energy flow distribution, energy loss density of unit structure, and overall structure energy loss density at 10 GHz.

Figure 4a presents the microwave reflectivity simulation curve in the 2–18 GHz band, and the results show that the microwave reflectivity in the X‐band is lower than 10%, realizing efficient absorption in this band. Figure 4b shows the simulation results of microwave reflectivity at different incident angles 0°, 20°, 40°, and 60°. At an oblique incidence angle of 60°, the X‐band microwave reflectivity can still be steadily kept below 15%, presenting excellent oblique incidence adaptability. Figure 4c shows the simulated electric field distribution and energy loss of the OMAL at the 10 GHz frequency point. It can be intuitively observed that the upper FSS and double‐layer ITO square ring structure possess high electric field density, and resonant current forms between the upper and lower film layers. Energy flow and loss are mainly concentrated inside the double‐layer ITO square ring structure. This result fully verifies the synergistic mechanism of FSS wave transmission and ITO absorption under both TE and TM modes, indicating that electromagnetic waves can efficiently pass through the FSS structure and achieve accurate absorption and energy dissipation at the double‐layer ITO square ring. The double‐layer ITO square ring structure effectively reduces the polarization sensitivity under large oblique incidence. Both TE and TM polarizations maintain stable absorption performance in the 0°–60° incident range, which lays a foundation for practical engineering applications.

To verify the process compatibility between the OMAL and the underlying VCL–WLSL composite structure, the influence of the FSS etching process on the laser stealth performance of the WLSL was analyzed. Affected by the skin depth effect, the 200 nm‐thick Ag layer at the bottom of the WLSL exhibits total reflection characteristics for X‐band microwaves, so the FSS pattern must be etched through the entire VCL and WLSL to form through gaps. According to the structural parameters, the area ratio of the etched hollow gaps is calculated as (*p*_1_^2^–*d*_1_^2^)/*p*_1_^2^ ≈ 13%, while the intact resonant cavity region of the unetched WLSL accounts for more than 87% of the total area and acts as the absolutely dominant part of the structure.

For the incidence of 10.6 μm laser within the range of 0°–60°, the vast majority of the laser energy is directly irradiated on the intact WLSL region to achieve efficient absorption. There is a three‐order‐of‐magnitude difference between the wavelength of the 10.6 μm laser and the feature size of the FSS. A small amount of energy may enter the etched hollow gaps, but it will be scattered in all directions and cannot form strong backward echo signals that can be received by the laser detection system. Therefore, the macroscopic optical response of the structure in the 10.6 μm laser band is completely dominated by the intact WLSL functional region with an absolute area advantage, and the etching fabrication of the FSS will not damage the core wide‐angle laser stealth performance of the WLSL.

### 2.5 Experimental Setup

Based on the analysis of the simulation results, 300 mm × 300 mm large‐area samples were fabricated and experimental tests were carried out to further validate the simulation findings. The VCL and WLSL were sequentially deposited on 0.05 mm‐thick PI substrates via electron beam evaporation with a vacuum degree below 5 × 10^−4^ Pa. The FSS structure was subsequently formed through femtosecond laser etching to ensure complete penetration of the VCL and WLSL without damaging the underlying structure. Visible‐light spectral measurements were performed using a Perkin Elmer LAMBDA 1050+ UV‐VIS‐NIR reflectance spectrometer, and the CIE 1931 chromaticity coordinates were calculated according to the standard colorimetric method. Fourier transform infrared spectral tests were conducted using a Bruker VERTEX 70 spectrometer with a spectral resolution of 4 cm^−1^, and a gold mirror was used as the reference for reflectivity calibration. Since the wide‐angle laser reflectivity is difficult to measure directly using a conventional FTIR spectrometer, a self‐built 10.6 μm CO_2_ laser test system was adopted, and the reflectivity was calculated by comparing the reflected power of the sample with that of a standard gold mirror, as presented in Equation (4). Figure 5 shows the experimental test environment, as‐fabricated samples and SEM images of the samples.

(4) $$R_{\text{Laser}}=P_{1}/P\times 100\%$$

where $R_{\text{Laser}}$ is the laser reflectivity, $P_{1}$ is the power energy received by the power meter for sample reflection, and P is the energy received by the power meter for total reflection.

**FIGURE 5:**
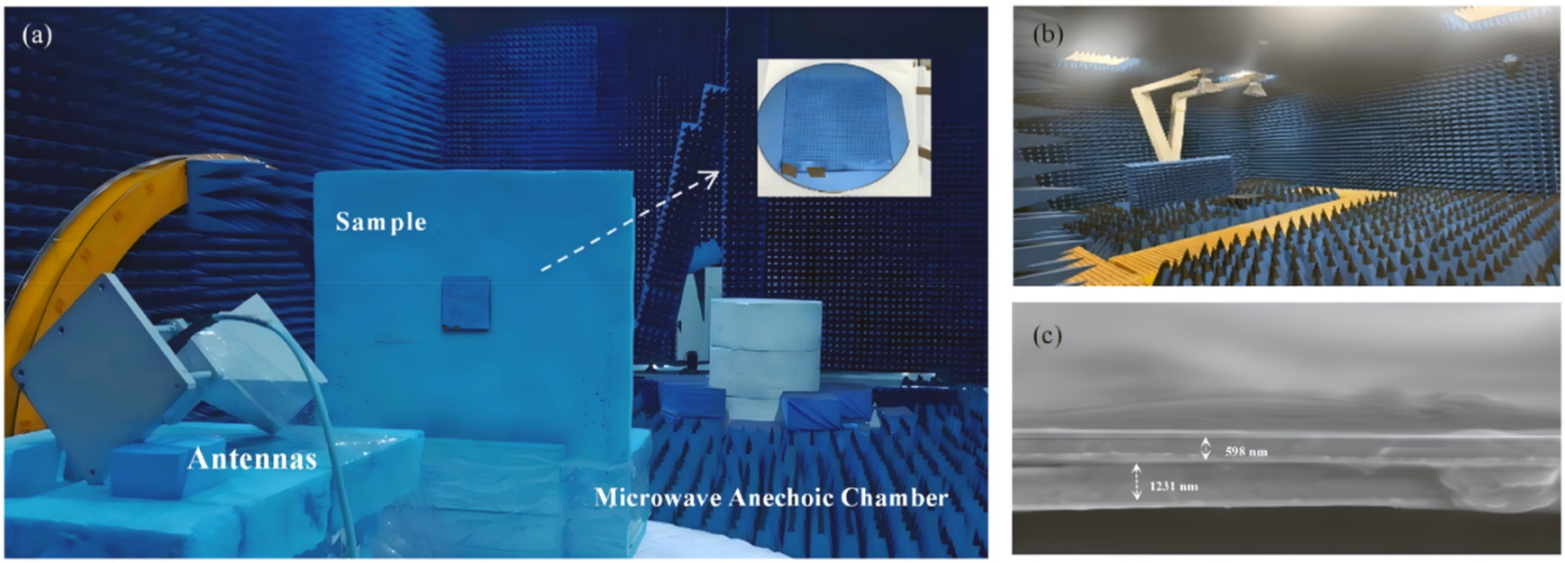
Experimental test. (a) The experimental test environment and as‐fabricated samples. (b) The microwave arch test fixture. (c) SEM images of the samples.

Figures 5a and 6a present the physical color and the measured CIE 1931 chromaticity coordinates of the samples, respectively, and the measured results are basically consistent with the simulation results. Figure 6b shows the measured infrared spectrum in the wavelength range of 5–16 μm. The measured reflectivity of the sample agrees well with the simulated trend. Owing to the thick metal layer, laser light can hardly penetrate the material, yielding an absorptivity over 90% at the 10.6 μm laser wavelength. Figure 6c displays the reflectivity characteristics of the 10.6 μm laser within the incident angle range of 0°–80°. The measured results indicate that the sample can meet the engineering threshold requirements for laser stealth within 0°–60° incidence, exhibiting excellent wide‐angle laser stealth performance. Figure 6d illustrates the measured microwave reflectivity mappings in the 2–18 GHz frequency band under 0°–70° oblique incidence for TE and TM polarizations, respectively. The test results show that within the 0°–60° incident range, X‐band microwaves maintain low reflectivity under both TE and TM polarizations, and the overall variation trend is in good agreement with the simulation results, verifying the stability of the wide‐angle microwave stealth performance. The minor numerical deviation between test data and simulation results mainly originates from the dimensional tolerance of laser‐etched FSS patterns, scattering loss caused by structural edge roughness, systematic calibration error of test instruments, and background clutter in the microwave anechoic chamber. The overall deviation is within the acceptable range for engineering verification and does not affect the judgment of core stealth performance.

**FIGURE 6:**
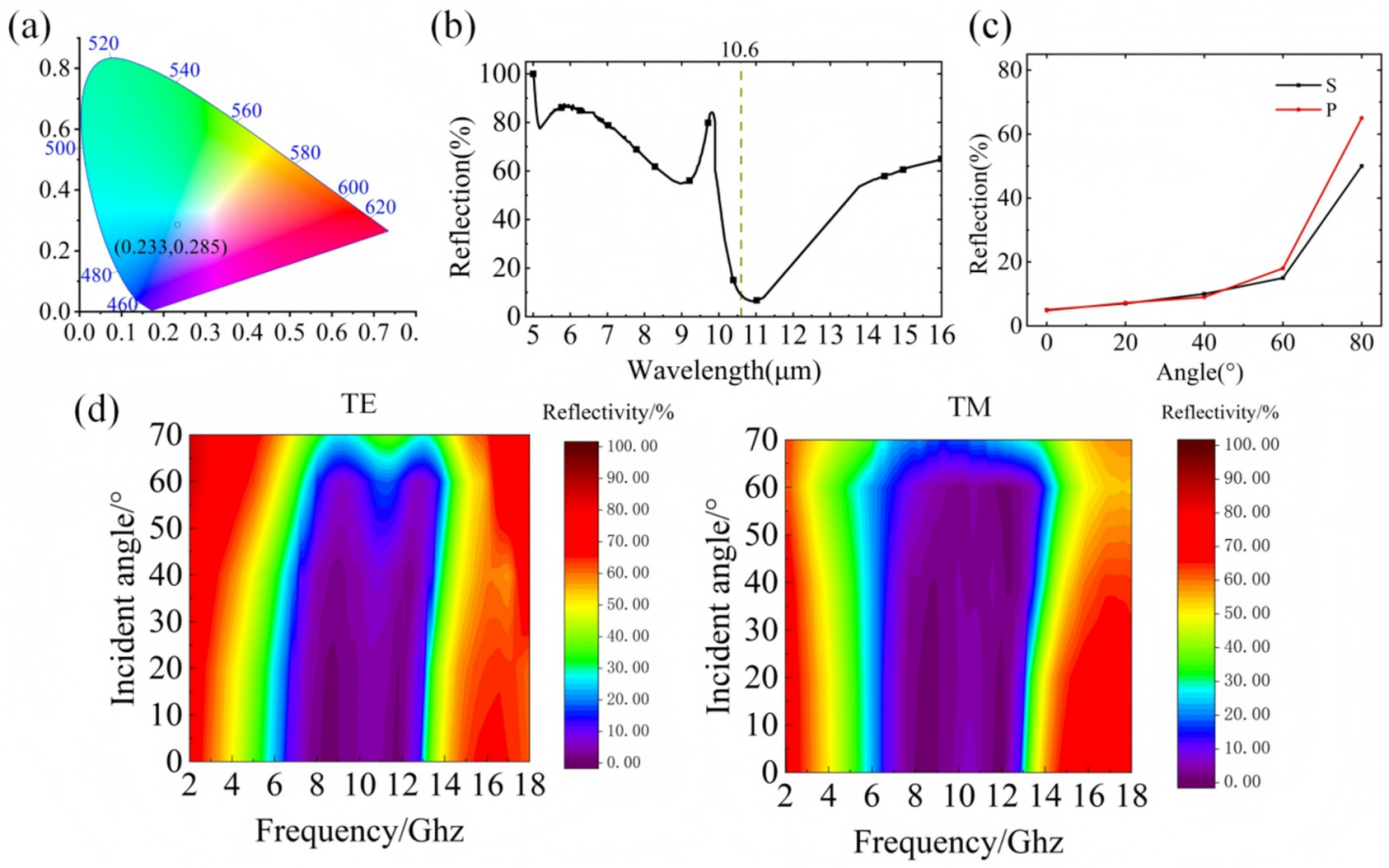
Test results. (a) Measured CIE 1931 chromaticity coordinates of samples. (b) Measured infrared spectrum ranging from 5 to 16 μm. (c) 10.6 μm laser reflectivity at incident angles of 0°–80°. (d) Measured microwave reflectivity mappings of TE and TM polarizations in 2–18 GHz under 0°–70° oblique incidence.

The comprehensive analysis and test of the stealth performance of the designed structure in each band show that the overall structure realizes the oblique incidence cooperative stealth of 10.6 μm laser and X‐band microwave through integrated coupling, and can realize independent decoupling and separate regulation of each band. The design process comprehensively considers the process feasibility and material flexibility, making the structure have high engineering realizability.

## 3 Conclusion

Aiming at the three core bottlenecks of conventional multispectral stealth technologies—including severe performance degradation under oblique incidence, multi‐band functional conflict, and poor conformal adaptability—this paper proposes a wide‐angle‐ compatible, flexible multispectral stealth metasurface, and systematically verifies its performance through theoretical analysis, simulation calculation, and experimental testing. Based on the order‐of‐magnitude difference in wavelength scale among different wavebands, the structure realizes functional synergy and independent decoupling regulation for each waveband. Specifically, the VCL achieves precise color matching with three typical natural backgrounds. The WLSL based on resonance pre‐biasing compensation realizes stable absorption of 10.6 μm laser in the 0°–60° incident range. The OMAL, based on high‐duty‐cycle FSS and double‐layer ITO square ring structure, achieves efficient absorption of X‐band microwaves under 60° oblique incidence. The test results of each waveband are in good agreement with the simulation results, which verifies the correctness of the design. Meanwhile, from material system selection to process route design, the feasibility for engineering implementation has been fully considered. The structure possesses flexible substrates and potential for conformal applications, and has technical potential for batch manufacturing and cost optimization. This work provides a new technical approach for solving the angle sensitivity problem of multispectral stealth, and has broad application prospects in the fields of photoelectric detection protection, target stealth, and electromagnetic compatibility.

## 4 Materials and Methods

### 4.1 Material Simulation and Testing

Optical simulations were performed using TFCalc software platforms, whereas microwave simulations were conducted via CST Microwave Studio. All experimental procedures were carried out within a microwave anechoic chamber. The samples were prepared by depositing thin films onto commercial PI substrates via electron beam evaporation, followed by laser micromachining for structural patterning. Visible and near‐IR spectral characterizations were conducted using a Perkin Elmer LAMBDA 1050+ UV‐Vis‐NIR spectrophotometer (Agilent Technologies, USA) at room temperature. IR spectra (5–14 μm) were acquired using a Bruker VERTEX 70 Fourier‐transform IR spectrometer. Microwave characterizations were measured and analyzed using an Agilent N5224 A vector network analyzer.

### 4.2 Material Fabrication

Multilayer thin films were deposited onto 100 × 100 mm PI substrates via electron beam evaporation in a high‐vacuum system (base pressure ≤ 5 × 10^−6^ Torr). Bulk ceramic targets were used as evaporation sources for SiO_2_ and ZnS, while elemental silver was melted in a molybdenum crucible under electron beam bombardment. Prior to deposition, substrates were treated with an ion source plasma cleaning process for 10 min to remove surface contaminants. Film thickness and growth rate were precisely controlled using a 360‐series quartz crystal thickness monitor, with the substrate holder rotating at 30 revolutions per minute to ensure uniform film coverage. To prevent thermal‐induced sulfidation of the Ag layer, the deposition process was conducted at room temperature, with evaporation parameters optimized for each layer to achieve the designed optical properties.

In the laboratory stage, electron beam evaporation and femtosecond laser etching are adopted to ensure nanoscale film thickness accuracy and micro‐nano pattern quality, meeting the high‐precision requirements of principle verification, which is not a process scheme for mass production. For large‐scale engineering applications, the structure is compatible with mature manufacturing processes such as magnetron sputtering for large‐area film deposition, and UV lithography combined with wet etching for batch patterning. In addition, PI/PET flexible substrates are compatible with roll‐to‐roll production modes, endowing the structure with technical potential for batch manufacturing and cost optimization.

## Author Contributions

L. Wang, C.L. Xu, Q. Fan and J.F. Wang conceived the idea and conducted theoretical analysis. J. Liu, J. Wang and S. Sui provided helpful advice to the work. L. Wang, C.L. Xu, Z.T. Chu and H. Yang carried out the simulation of the designed metasurface. H. Yang, J.M. Jiang and Y.X. Jia conducted experimental tests. L. Wang, C.L. Xu, Q. Fan and J.F. Wang participated in all the data analysis. L. Wang, C.L. Xu, Q. Fan and J.F. Wang wrote the paper, and all authors revised the paper.

## Conflicts of Interest

The authors declare no conflicts of interest.

## Data Availability

The data that support the findings of this study are available from the corresponding author upon reasonable request.
